# The prevalence of non-cardiac chest pain (NCCP) using emergency department (ED) data: a Northern Ireland based study

**DOI:** 10.1186/s12913-017-2493-8

**Published:** 2017-08-09

**Authors:** Orla McDevitt-Petrovic, Karen Kirby, Mark Shevlin

**Affiliations:** School of Psychology and Psychology research Institute, Ulster University, Derry, BT48 7JL UK

## Abstract

**Background:**

The aim of this study was to assess the frequency of chest pain presentations and the subsequent non-cardiac chest pain diagnoses in an emergency department (ED) over a 3 year period.

**Methods:**

Administrative data on ED attendances to an urban general hospital in Northern Ireland between March 2013 and March 2016 were used. Data were coded and analysed to estimate frequencies of ‘chest pain’ presentation and the subsequent diagnoses for each year.

**Results:**

Both chest pain presentations and chest pain presentations with a subsequent diagnosis of unknown cause increased each year. In total, 58.7% of all chest presentations across 3 years resulted in a non-cardiac diagnosis of either ‘anxiety’, ‘panic’ or ‘chest pain of unknown cause’.

**Discussion:**

There is a significant amount of patients in the ED leaving with a non-cardiac diagnosis, following an initial presentation with chest pain.

**Conclusion:**

Given the link between non-cardiac chest pain and frequent use of services, the degree of repeat attendance should be investigated.

## Background

Chest pain is a common complaint that is frequently non-cardiac in origin. Research to date indicates that between 52 and 77% of chest pain presentations to accident and emergency departments (ED) are discharged without a clear medical diagnosis, and thereby may be referred to as incidences of unexplained chest pain or non-cardiac chest pain (NCCP**)** [1–3]. A recent review of clinical care posited that focus is more often on the exclusion of medical diagnosis and not on the appropriate management of NCCP. Consequently, NCCP has been identified as a significant public health concern linked with limitation of activities, absenteeism, unemployment, disability, and repeated attendance at EDs, primary care, and acute services [4].

### Importance

Frequent users constitute 4.5 to 8% of all ED patients, however, they represent 21 to 28% of all visits to the ED where frequent is defined as four or more visits in a year [5]. Chest pain accounts for 21% of primary presentations from 1200 visits [6]. However, strategies to redirect patients from emergency care are often rejected, given that many visits are considered necessary based on the presenting symptoms [5]. In the U.S., costs for the initial care for NCCP has been estimated at eight billion dollars although limited information is available on the long term economic consequences of NCCP [7].

### The role of anxiety in non-cardiac chest pain

Anxiety has a key role in the neuro-behavioural processes associated with pain regulation, possibly contributing to NCCP. Some of the autonomic symptoms of anxiety are also features of NCCP, which may explain why higher levels of anxiety have been detected among individuals with NCCP compared with healthy individuals [8]. This is also consistent with previous research that has indicated that the prevalence of panic disorder in NCCP is significantly higher than in the general population [9, 10].

The prevalence rates of anxiety and depression are similar among individuals with an actual cardiac disease diagnosis and those identified as presenting with NCCP [4]. Importantly however, research showed that these psychological difficulties are much less inclined to be resolved in NCCP [11–13] and are also associated with more heath related cognitive distortions [14]. Despite reassurance by emergency physicians and cardiologists, more than 50% of patients presenting with NCCP continue to report chest discomfort and remain worried they have a serious health condition or have suffered myocardial infarction [15].

A high prevalence (44%) of panic-like anxiety has been found in ED presentations, using valid psychometric assessments, however it was also found that physicians in EDs diagnosed just 7.4% of these panic cases [16]. In addition to this, studies have highlighted that even where panic and anxiety are identified in the emergency department the appropriate interventions are seldom initiated within the scope of these services [17, 18]. It has been suggested that the need to increase understanding of NCCP in emergency departments is critical, and that improved access to the appropriate psychological interventions could reduce attendance at health services and limit the social, physical, and occupational costs associated with anxiety and panic disorders [16].

### Goals of this investigation

Evidence has shown that a significant proportion of admissions to the ED presenting with chest pains will result in a diagnosis of NCCP, however there is scarce data on the frequency of presenting and diagnoses in the U.K. Based on the recommendations of existing research, and following the requests of physicians in a UK hospital, it was considered important to assess the frequency of NCCP cases presenting in ED departments in Northern Ireland, as to date no such studies have been carried out. The outcome of such a study would support the need (or not as the case may be) to provide additional psychological services for people who are repeatedly using hospital services for NCCP with possible associated anxiety or panic disorders. The primary aim of this study aimed was to use administrative data to assess the number of chest pain admissions to an ED department and the subsequent counts of NCCP over a 3 year period. The likelihood of NCCP based on admission with chest pain was estimated.

## Method

### Study design and data access

This study used administrative data from an urban district general hospital in the North West of Northern Ireland. Records relating to initial presentation and subsequent diagnosis for 3 years were analysed. With the permission of service managers within Clinical Governance and the Western Trust Service Improvement Department, records for all patients attending the ED between March 2013 and March 2016 (*N* = 180, 409) were accessed by Trust administrative staff. All entries were anonymised. Data for each year was examined in order to 1) assess the frequency of attending the ED with chest pain or heart problems, 2) assess the frequency of diagnoses related to NCCP, anxiety and panic, and 3) to estimate the relationship between presenting with chest/heart related problems and a subsequent diagnosis of NCCP or anxiety.

### Data management and analysis procedures

The following strategies were implemented in order to estimate the frequency of attending the ED with chest pain and heart problems, and to calculate the counts of diagnoses related to NCCP, anxiety and panic.

Stage 1: There was no standardised format for recording specific presenting problem. The research team initially reviewed the entries for each year to identify potential ways in which ‘heart related’ problems were recorded. A systematic review of the entries revealed that the most common descriptors were ‘chest pain’, ‘palpitation (s)’, ‘heart’, and ‘chest tightness’. These were subsequently used as search terms to identify those attending ED with ‘heart related problems’.

Stage 2: There was no standardised format for recording diagnoses. A systematic review of entries identified the most common descriptor of non-cardiac diagnosis to be ‘chest pain of unknown cause.’ Three columns were created for diagnoses, with ‘anxiety’, ‘panic’ and ‘chest pain of unknown cause’ coded as 1, and all other ‘non’ cases within these were coded as 0.

Stage 3: Frequencies and percentages of chest pain presentations and specific non-cardiac diagnosis for each year (2013/14, 2014/15, and 2015/16) were calculated (see Table 1), in addition to frequencies and percentages of chest pain presentations and specific non-cardiac diagnosis and any non-cardiac diagnosis for all years combined (see Tables 2 and 3). Chi-square tests were carried out in order to estimate the degree of association of non-cardiac diagnoses and all other diagnoses within chest pain presentations across the total sample (see Tables 1 and 2).

**Table 1:** Frequencies and percentages of chest pain presentations and specific non-cardiac diagnosis for each year (2013/14, 2014/15, and 2015/16)

Chest pain presentations and corresponding diagnoses			Year(s)		Total	
			2013/14 (*N* = 59,244)	2014/15 (*N* = 58,123)	2015/16 (*N* = 63,122)	2013-2016 (*N* = 180, 489)
Presenting with chest pain	Yes	N	2381	2675	3239	8295
		%	4.1%	4.6%	5.1%	4.6%
	No	N	56,863	55,448	59,883	172,194
		%	95.9%	95.4%	94.9%	95.4%
			Presenting with chest pain 2013/14 (*N* = 2, 381)	Presenting with chest pain 2014/15 (*N* = 2, 675)	Presenting with chest pain 2015/16 (*N* = 3, 239)	Presenting with chest pain 2013-2016 (*N* = 8, 295)
Diagnosis of ‘anxiety’ within chest pain presentations	Yes	N	56	34	45	135
		%	2.4%	1.3%	1.4%	1.6%
	No	N	2325	2641	3194	8160
		%	97.6%	97.7%	98.6%	98.4%
Diagnosis of ‘panic’ within chest pain presentations	Yes	N	13	20	21	54
		%	0.5%	0.75%	0.6%	0.6%
	No	N	2368	2655	3218	180,435
		%	99.5%	99.25%	99.4%	99.4%
Diagnosis ‘Chest pain of unknown cause’ within chest pain presentations	Yes	N	804	1049	1303	3156
		%	33.8%	39.2%	40.2%	38%
	No	N	1577	1626	1936	5139
		%	66.2%	60.8%	59.8%	62%

**Table 2:** Frequencies and percentages of chest pain presentation and specific diagnoses for all years

			Diagnosis						Total	Diagnosis	
			No Anxiety (*N* = 179,902)	Anxiety (*N* = 551)	No Panic (*N* = 180,223)	Panic (*N* = 220)	No CPUC (*N* = 175,525)	CPUC (*N* = 4929)		All other Diagnoses (*N* = 174,753)	^a^Any Non-Cardiac Diagnosis (*N* = 5700)
Presenting with chest pain	Yes	N	8160	135	8241	54	5139	3156	8295	4950	3345
		%	4.5%	24.5%	4.6%	24.5%	2.9%	64%	4.6%	2.8%	58.7%
	No	N	171,742	416	171,992	166	170,386	1773	172,158	169,803	2355
		%	95.5%	75.5%	95.4%	75.5%	97.1%	36%	95.4%	97.2%	41.3%
			χ^2^(1) = 499.29, *p* < .001		χ^2^(1) = 199.88, *p* < .001		χ^2^(1) = 40,815.10, *p* < .001			χ^2^(1) = 39,263.76, *p* < .001	

*CPUC* Chest pain of unknown cause

^a^Indicates all cases discharged with anxiety, panic or CPUC

Stage 4: For the purposes of inter-rater reliability, a second researcher also completed a systematic review of entries as outlined in stages one and two. The identified terms were then used and subsequently the second researcher determined the same frequencies as the first researcher. The results obtained were therefore identical for both researchers.

## Results

Table 1 shows that there was an annual increase in the overall number of ED attendances during the period 2013 to 2016, increasing from 59,244 to 63,122. There was a corresponding increase in the frequency of patients presenting with chest pain (4.1 to 5.1%) over the same period, and chest pain presentations with a subsequent diagnosis of unknown cause similarly increased year on year. The most common non-cardiac diagnosis each year was ‘Chest pain of unknown cause’ (33.8 - 40.2%), with a relatively small number of diagnoses of ‘anxiety’ (1.3 - 2.4%) or ‘panic’ (.5 - .75%).

Table 2 shows that when all 3 years were combined 8295 (4.6%) of initial presentations were chest pain, and 135 (24.4%) and 54 (24.5%) were anxiety and panic diagnoses respectively following an initial presentation of chest pain. Over half (*n* = 3156, 64%) of diagnoses indicating ‘chest pain of unknown cause’ followed an initial presentation of chest pain. The chi-square tests showed that presenting with chest pains and receiving a diagnosis of panic, anxiety, or CPUC were significantly associated. In total, 3345 (58.7%) of all the non-cardiac diagnoses (anxiety, panic and chest pain of unknown cause) followed an initial presentation with chest pain, and this association was statistically significant (χ^2^ (1) = 39,263.76, *p* < .001).

## Discussion

The findings from this study are consistent with those in the current literature [1–3, 19] given that a large proportion of patients presenting at ED with ‘chest pains’ were subsequently given a non-cardiac diagnosis: anxiety (1.6%), panic (0.6%), or NCCP (38%). This pattern of results was consistent over the 3 years of this study. Little is known about the long term economic costs of NCCP, however studies to date have highlighted that costs to services are likely to be substantial [7]. NCCP patients have been found to use out-patient services to the same extent as those who have cardiac diagnosis [15]. Although less is known about the indirect costs, a recent longitudinal study concluded that both the direct healthcare, and cumulative societal costs of NCCP are considerable [20].

### What is the prognosis for patients with NCCP?

Although the prognosis may not be concerning in medical terms, it has been determined that NCCP patients frequently endure chronic symptoms in addition to high levels of psychological distress. Up to 50% of NCCP remain worried they have a serious condition and continue to needlessly use cardiac services despite reassurance from physicians [15]. Research furthermore indicates that psychological difficulties are much less inclined to be resolved in NCCP compared with actual cardiac diagnosis [4].

It has also been suggested that anxiety is extremely prevalent, and yet insufficiently recognised and managed within emergency care settings [21]. A number of possible reasons have been suggested for this, including the hesitation of physicians to enquire, given that patients can be defensive about potential psychological causes [22]. Based on the findings of the current study it may be suggested that anxiety may not have been picked up as it may not have been screened for or assessed for after a cardiac diagnosis has been ruled out. It has also been established that even where panic and anxiety are recognised within the emergency care services, the appropriate interventions are seldom initiated there [17, 18].

### Interventions

Current reviews of clinical care have highlighted a failure to appropriately manage NCCP despite the substantial prevalence rates [4]. Studies to date have pointed to the efficacy of cognitive behavioural therapy (CBT) [23, 24], and the efficacy of CBT as an intervention for NCCP has been evaluated in a number of randomised controlled trials [25]. NCCP patients who had completed a course of CBT had a significantly higher treatment response when compared with placebo and medication groups [23]. Low intensity CBT intervention, more specifically “coping skills” resulted in significant improvement relating to the catastrophizing of pain symptoms and anxiety when compared to a placebo group [26].

Recent research has also emphasised the success of brief cognitive behavioural therapy; a three session CBT intervention was found to be effective for NCCP patients in terms of illness perception [27]. A brief cognitive behavioural intervention significantly reduced levels of anxiety and depression in patients with NCCP, with a diagnosis of panic and/or a depressive disorder [15], and cognitive behavioural interventions as brief as even a single session initiated within 2 weeks of an emergency attendance for the primary complaint of chest pain, seem to be effective for panic disorder [28].

Several studies have shown that NCCP patients frequently endure chronic physical symptoms and high levels of psychological distress [14, 29]. Given the high percentage of patients in the current study who were discharged with an outcome of ‘chest pain of unknown cause’, future work is needed to assess the degree and prevalence of anxiety disorders among this group in Northern Ireland. Furthermore, given the evidence base for the efficacy of CBT within this patient population, it may be posited that future research is also warranted to evaluate the effectiveness of a CBT intervention for those patients identified as having NCCP and associated significant anxiety.

### Limitations

It is important to note that the data used to obtain frequencies in the current study was fully anonymised and indicated only primary presenting problem and the subsequent diagnosis. It was not possible to determine if the same individual had attended services with the same or a similar presentation more than once. This is an acknowledged limitation of the current study but it can also be a future research recommendation to attempt to track repeat attenders who were discharged with NCCP. The non-standardised format of recording presenting problems was also a limitation. However, a thorough systematic review of entries was conducted and inter-rater reliability was established. It is also important to acknowledge the limitations relating to generalizability, given that the data pertains to a single emergency department.

### Practical and clinical research recommendations

In order to improve the recording of patient data on the database it would be useful to implement a standardised system to record presentations and outcomes.

Given the association between NCCP and frequent and inappropriate use of services, and the costs to healthcare systems, the degree of repeat attendance should be investigated. There is a need to determine what the current communication and referral pathways are for individuals with NCCP, and to actually assess and determine the views and experiences of individuals who are NCCP repeat attenders. The association between NCCP and clinical anxiety warrants further exploration, more specifically in relation to the prevalence of anxiety among this patient population, and the development of appropriate and timely psychological interventions. A more detailed psychosocial and medical profile of those who repeatedly attend emergency services with non-cardiac chest pain, would help in identifying other potentially significant associated factors.

Future research may take the form of a randomised controlled trial facilitating the piloting of an intervention. The first element would provide NCCP patients with some type of psychoeducational material regarding the nature of anxiety symptoms. The second element would involve setting up a referral pathway from emergency department staff to the GP. The GP would subsequently refer the patient to the psychological therapies hub from which onward referral to the appropriate service would be made. Governments are increasingly recognising the long term economic and social costs of high prevalence disorders including anxiety and panic disorder. Low intensity cognitive behavioural therapy is the most strongly evidenced in relation to common anxiety disorders, and moreover it has been shown to save money in the long term [30]. It is still the case however, that we do not have established care pathways in Northern Ireland which suitably apply the evidence, in order to facilitate the provision of timely and appropriate interventions [30].

A final recommendation relates to the provision of additional training for emergency department staff particularly in relation to accurately identifying these individuals and how to subsequently refer them to an appropriate service.

## Conclusion

This study has shown that there is a significant percentage of patients attending the ED with NCCP. Given the link between NCCP and frequent and inappropriate use of services, and the costs to healthcare systems, the degree of repeat attendance should be investigated. Furthermore, the association between NCCP and clinical anxiety warrants further exploration, more specifically in relation to the prevalence of anxiety among this patient population, and the development of appropriate and timely psychological interventions.
